# Experimental Demonstration of MmWave Vehicle-to-Vehicle Communications Using IEEE 802.11ad

**DOI:** 10.3390/s19092057

**Published:** 2019-05-02

**Authors:** Wooseong Kim

**Affiliations:** Department of computer engineering, Gachon University, Seongnam 13120, Korea; wooseong@gachon.ac.kr

**Keywords:** vehicular communications, mmWave spectrum, WiGig

## Abstract

Millimeter wave (mmWave) vehicle-to-vehicle (V2V) communications has received significant attention as one of the key applications in 5G technology, which is called as Giga-V2V (GiV2V). The ultra-wide band of the GiV2V allows vehicles to transfer gigabit data within a few seconds, which can achieve platooning of autonomous vehicles. The platooning process requires the rich data of a 4K dash-camera and LiDAR sensors for accurate vehicle control. To achieve this, 3GPP, a global organization of standards that provides specifications for the 5G mobile technology, is developing a new standard for GiV2V technology by extending the existing specification for device-to-device (D2D) communication. Meanwhile, in the last decade, the mmWave spectrum has been used in the wireless local area network (WLAN) for indoor devices, such as home appliances, based on the IEEE 802.11ad (also known as Wireless Gigabit Alliance (WiGig)) technology, which supports gigabit wireless connectivity of approximately 10 m distance in the 60-GHz frequency spectrum. The WiGig technology has been commercialized and used for various applications ranging from Internet access points to set-top boxes for televisions. In this study, we investigated the applicability of the WiGig technology to the GiV2V communications through experiments on a real vehicular testbed. To achieve this, we built a testbed using commercial off-the-shelf WiGig devices and performed experiments to measure inter-vehicle connectivity on a campus and on city roads with different permitted vehicle speeds. The experimental results demonstrate that disconnections occurred frequently due to the short radio range and the connectivity varied with the vehicle speed. However, the instantaneous throughput was sufficient to exchange large data between moving vehicles in different road environments.

## 1. Introduction

Various 5G wireless technologies were standardized and developed by academia and industries related to the field of telecommunications, and are now ready to be deployed with several key features such as enhanced mobile broadband (eMBB), ultra reliable low latency communications (URLLC), etc. A new radio for the 5G wireless networks is expected to use various radio frequencies, above or below 6 GHz. As compared to the 28, 60, and 73 GHz spectrums, the frequencies near the 3 GHz spectrum receive considerable attention from service providers because the millimeter Wave (mmWave) suffers from significant pathloss and link blockage caused by obstacles (e.g., buildings, vehicles and human beings) owing to severe penetration loss and reflection. However, it is advantageous to still consider the mmWave spectrum to obtain several hundred mega-hertz (MHz) of bandwidth because of the scarcity of spectrum below 6 GHz. Thus, measurement campaigns and demonstrations of mmWave communications have been conducted in real testbeds [1,2,3]. Subsequently, mmWave channel models are established based on these measurement results [4,5,6,7].

The 5G era has reserved the development of various applications and services for the near future as they require gigabit data rate, fast response (less than 1 ms for end-to-end delay), and massive connections. One such interesting application is vehicular communication for autonomous driving and in-vehicle infotainment. Autonomous driving systems primarily rely on sensor devices equipped on vehicles, such as LiDAR, near-medium range radar, long-range radar (LRR) and cameras. However, communications between vehicles or a vehicle and an infrastructure such as a road-side unit (RSU) are essential to control a fleet of vehicles to avoid chain-reaction crashes instead of controlling individual cars. Moreover, an autonomous vehicle has rich data, such as high-resolution video (e.g., UHD 4K) from dash cameras, which can be used to improve vehicle control precisely. For instance, the curvature recognition of the road in front of the vehicle can be improved from 30 m to 50 m in distance ahead if using 2 MP instead of 0.3 MP data [8].

In the last decade, dedicated short-range communications (DSRC) and IEEE 802.11p/wireless access in vehicular environment (WAVE) standards have guided vehicle-to-vehicle (V2V) communications, which enables vehicles to exchange safety messages to alarm or notify vehicle status and infotainment data using a 10 MHz bandwidth channel that supports up to 27 Mbps data rate. Further, the third generation partnership program (3GPP) introduced long-term evolution (LTE) based vehicle to infrastructure (V2I) communication and device-to-device (D2D) communication for the V2V communication. However, neither technologies is sufficient to transmit the increasing sensor data of future smart cars. Accordingly, 3GPP [9] is developing a new standard applying the 5G radio (e.g., mmWave) to vehicular communications in Release 19. Recently, various academic studies on mmWave-based vehicle-to-everything (V2X) communications were conducted. Practical mmWave vehicular testbeds have been built to evaluate the performance of V2I or V2V [10,11]. Moreover, the channel models of the V2X communications are exploited in [12,13,14,15] and several ideas to reduce beam alignment and training overhead are proposed in [16,17,18,19]. The problems of mmWave beam alignment and width selection to reduce interference among vehicles are solved by a distributed algorithm in [20,21,22]. Kim [8] proposed a channel assignment algorithm for mmWave V2V beams to avoid reciprocal beam interference.

The IEEE 802.11ad standard, also known as the Wireless Gigabit Alliance (WiGig), which specifies the physical and link layers for mmWave communications, was released approximately a decade ago to provide a gigabit connection between proximate devices. The WiGig uses a 60 GHz mmWave spectrum and supports more than 4 Gbps data rate with 2 GHz channel bandwidth. Furthermore, indirect communication using a relay node is possible when a link blockage occurs; a sending station chooses a relay node that has a line-of-sight (LOS) link to a receiver. In this study, we investigated the applicability of the IEEE 802.11ad standard to a gigabit V2V communication (GiV2V) system in a vehicular testbed, for which we utilized off-the-shelf device modules based on the IEEE 802.11ad standard and installed them on the roof of the vehicles. A LOS direct connection satisfying the IEEE 802.11ad requirements between two vehicles was configured and the throughput and connectivity were measured while driving on the roads. This measurement was performed on low-speed campus roads and high-speed city roads, respectively, to evaluate the effect of vehicle mobility. To the best of our knowledge, this study was the first attempt at the measurement of V2V communications using commercial IEEE 802.11ad modules.

The highlights of our contributions in this study are as follows:

- We conducted mmWave V2V communications using commercial IEEE 802.11ad modules.
- We analyzed inter-vehicle connectivity by mmWave of short-range radio.
- We compared the mmWave V2V communications in different driving environments.

According to our experimental results, the IEEE 802.11ad modules perform only in a short radio range of approximately 10–20 m at boresight due to significant pathloss, which causes frequent disconnections between vehicles, especially during high mobility. However, the average disconnection time is approximately 1 s, irrespective of the vehicle speed, even though the deviation of disconnection differs based on the speed; thus, high-speed vehicles demonstrate a larger deviation of disconnection time than low-speed vehicles. Regardless of the frequent disconnections, the experimental results demonstrate that the mmWave connectivity can transmit a large amount of data within a few seconds in the future smart cars.

The remainder of the paper is organized as follows. In Section 2, we introduce the background and related works on mmWave and vehicular communication technology. We describe the GiV2V system in Section 3. Section 4 describes our experimental configuration, followed by the results in Section 5. Finally, we discuss and conclude our study in Section 6.

## 2. Related Works

In the last decade, the mmWave spectrum was popularly explored to enlarge the bandwidth and increase the throughput in mobile communications for a last decade. Rappaport et al. introduced seminal results of the experiments using a wideband sliding correlator channel sounder with steerable directional horn antennas at both the transmitter and receiver in New York city in [1] from 2011 to 2013, and presented more results on the frequency bands of 28, 38, and 73 GHz bands in [2,3].

Based on those studies, mmWave channel models are demonstrated in [4,5], where empirically-based propagation channel models are proposed for the 28, 38, 60, and 73 GHz mmWave bands. Samimi et al. presented a 3-D statistical channel impulse response model for urban LOS and non-LOS (NLOS) channels developed from the 28 and 73 GHz ultra-wideband propagation measurements presented [6,7]. They later added small-scale fading measurements for the 28 GHz outdoor mmWave ultrawideband channels using directional horn antennas [23]. Additionally, 28 GHz wideband propagation channel characteristics for mmWave urban cellular communication systems are presented in [24].

Sun et al. characterized a mmWave indoor propagation channel based on a wideband measurement campaign at 73 GHz in an office-type environment [25]. For outdoor access channel modeling at 60 GHz mmWave spectrum, Weiler et al. [26] established a quasi-deterministic channel model and a link level-focused channel model and, subsequently, the authors of [27] introduced a new quasi-deterministic (Q-D) approach for modeling mmWave channels, which allows natural description of scenario-specific geometric properties, reflection attenuation and scattering, ray blockage, and mobility effects.

Andrews et al. provided a comprehensive overview of mathematical models and analytical techniques for mmWave cellular systems based on stochastic geometry [28]. In addition, a system-level analysis of the success probability for cell selection and random access delay in the mmWave cellular systems is conducted in [29].

Currently, the mmWave communication is now considered for V2V or V2I communications, which are required for autonomous or proxy driving in future smart cars [9]. We survey existing works related to the mmWave V2X communications as in Table 1.

Several studies on measurement campaigns and channel modeling for the mmWave V2V or V2I communications are reported. Ben-Dor et al. conducted multipath and angle-of-arrival (AOA) measurements at 60 GHz of outdoor peer-to-peer channels in an urban campus courtyard for transmission and into a vehicle [30] in 2011, demonstrating that varying the transmitter and receiver separation provides different root mean square (RMS) delay spreads from 2.73 ns to a maximum value of 12.3 ns for the LOS and NLOS antenna pointing scenarios. Loch et al. developed a practical mmWave vehicular testbed to evaluate performance, where fixed beam-steering approach enables the RSUs to transmit large amounts of data in a considerably short amount of time for a wide range of speeds [10]. Park et al. investigated mmWave blockage characteristics based on measurements collected in a typical V2V environment at 28 GHz [11].

Based on these measurements and simulations, the V2X channel models are exploited. Va et al. reviewed the state-of-the-art in measurements related to mmWave vehicular channels [12]. In contrast to the previous studies conducted with the two-ray model on flat road surfaces, more realistic settings with road undulation, road surface curvature, and blockage by other vehicles are considered to improve the accuracy of pathloss prediction. The propagation mechanisms reflection and diffraction of mmWave on realistic road surfaces and geometries at 60–77 GHz are demonstrated in [31,32]. Further, Antoescu et al. proposed channel propagation models for mmWave V2X communications using ray-tracing simulations [13], which include the effects of link blockage, scattering and multipath fading. Wang et al. provided research results on propagation characteristics of V2V channels, particularly for shadowing effects induced by obstructing vehicles between a transmitter and receiver [14]. In [15,33], a geometric multiple-input multiple-output (MIMO) channel model for mmWave mobile-to-mobile (M2M) applications based on the two-ring reference model is proposed.

Several studies on the V2X network based on a stochastic geometry model are reported that investigate and optimize the connectivity and throughput with varying blockage, beam direction and vehicle density. Tassi et al. proposed a stochastic model of mmWave-based RSUs infra-structure to vehicle communications [34], where they investigated the blockage probability and throughput with varying vehicle densities and speeds at a multi-lane highway. Lorca et al. presented a theoretical analysis of the Doppler power spectrum in the presence of beamforming at the transmitter and/or the receiver in V2I systems [35]. Wang et al. analyzed the coverage of urban mmWave micro-cellular networks based on stochastic geometry with a LOS probability function of randomly oriented buildings for a V2I scenario [36]. Perfecto et al. analyzed the interplay between the beamwidth assignment and the scheduling period in V2V communications [20] and proposed an optimization algorithm to establish a V2V link having optimal beam width, using swarm intelligence based on the channel and queue state information [21]. Va et al. proposed a swarm intelligence to efficiently pair vehicles of V2V links and optimize beam widths considering the channel state information and queue state information [22].

Some studies propose approaches to utilize sensors (such as radars, GPS, camera, etc.) to reduce the beam alignment overhead among vehicles based on vehicle position, posture, or other sensed information. Gonzalez et al. [19] and Kumari et al. [18] proposed a set of algorithms to perform the beam alignment in a V2I scenario, by extracting information from the IEEE 802.11ad module or the LRR radar signal to configure the beams or create a joint waveform for automotive radars and mmWave V2V communications using the same hardware. In [16], Choi et al. proposed a high-level solution for a key challenge of the mmWave beam training overhead where the information derived from the sensors or DSRC are leveraged as lateral information for the mmWave communication link configuration. Mavromatics et al. leveraged vehicle sensory data of position and the motion for beam forming by DSRC beacons [17].

Similarly, location-based beam alignment and training are considered in the following studies. Va et al. proposed a mmWave-beam switching approach based on the position information (for example, the information available via GPS) from the train control system for efficient beam alignment [37] and presented an optimization of beam design to maximize the data rate for non-overlap beams in the LOS to the RSU [38]. Garcia et al. also proposed a location-aided beam forming strategy and analyzed the resulting performance considering the antenna gain and latency [39]. Maschietti et al. formulated the optimum beam alignment solution of a Bayesian team decision problem with novel and less complicated algorithms for optimality [40]. Va et al. leveraged the position of a vehicle along the past beam measurements to rank desirable pointing directions that can reduce the required beam training based on a popular machine learning method used in recommender systems [41]; moreover, they proposed the utilization of the position of the vehicle to query a multipath fingerprint database that provides prior knowledge of potential pointing directions for reliable beam alignment [42]. In [43], Wang et al. also introduced machine learning with the past beam training records for optimal beam pairing by exploiting the locations and sizes of the receiver and its neighboring vehicles.

Eltayeb et al. proposed a blockage detection technique for mmWave vehicular antenna arrays that jointly estimate the locations of the blocked antennas along with the attenuation and phase-shifts that result from the suspended particles [44]. In such a blockage, a joint optimization problem to select a relay and link to circumvent obstacles and to reduce delivery latency in the 60 GHz mmWave networks was modeled by He et al. [45] together with a less complex algorithm decomposing the problem into tractable sub-problems. Furthermore, Taya et al. proposed a multi-hop relaying through dynamic vehicle deployment to increase the coverage of V2X, formulate the deployment problem as an optimization problem, and obtain its lower and upper bounds of performances [46].

In [47], Petrov et al. showed that the interference from the adjacent lanes can be reasonably approximated using two-dimensional stochastic models without any significant loss of accuracy. This interference may significantly affect the performance of the communication systems as highly directional antennas are used by spatial configurations. Kim et al. proposed a channel assignment algorithm for mmWave beams to avoid the inter-beam interference from the uncoordinated beams in mmWave V2V communications [8].

Multiple connections to legacy network (such as 3G or LTE) and mmWave base stations can provide seamless connectivity to moving vehicles. Giordani et al. introduced a method with multi-connectivity to a mmWave cell and a conventional microwave cell for robust connectivity and handover based on a sounding signal sweeping and instantaneous measurement of the received signal strength measurement [48]; further, they proposed a novel uplink multi-connectivity system for the efficient control plane applications, such as handover, beam tracking, and initial access [49].

For security in vehicular mmWave communication systems, Eltayeb et al. proposed physical layer security techniques by injecting artificial noise in controlled directions using multiple antennas [50].

These previous studies on the vehicular communications attempt to establish a vehicular channel model of mmWave V2V or V2I communications through simulation or mathematical modeling and propose ideas to reduce beam alignment overhead and training based on location or sensor data. In this study, we demonstrated the feasibility of V2V communication using the IEEE 802.11ad with the 60 GHz mmWave spectrum, specifically in a LOS environment. For the IEEE 802.11ad standard, Jacob et al. explored a channel model for system level simulations with medium access control (MAC) protocols to investigate the influence of moving humans in the framework of IEEE 802.11ad standard [51]. Coll et al. evaluated the IEEE 802.11ad standard for V2V communication through simulation [52], demonstrating that the MAC operation and beamforming processes result in a high overhead, and the uncoordinated transmitting stations can significantly degrade network throughput. However, to the best of our knowledge, there has been no experimental study with off-the-shelf IEEE 802.11ad devices in a driving testbed.

## 3. GiV2V Communication Architecture

**Figure 1:**
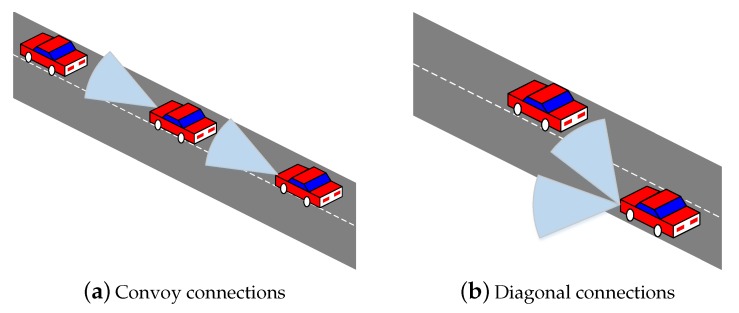
GiV2V topology.

In this section, we introduce the GiV2V architecture and beam forming process according to the GiV2V topologies. Further, a well-known pathloss model of the IEEE 802.11ad standard is introduced to verify the applicability of this pathloss model to our measurement results in Section 4. The GiV2V network topology is shown in Figure 1. The V2V communications for autonomous driving occur typically in a vehicular platoon, as illustrated in Figure 1a, in which a single driver or the front car leads multiple trailing vehicles similar to a train. In this convoy model, the GiV2V can form a front or rear beam to obtain directivity gain in mmWave communications. Thus, the connectivity between vehicles can be more stable when compared to other traffic formations as the beam direction is unchanged and inter-vehicle distance can be maintained consistently by controlling vehicle speed. In other vehicle topologies, vehicles require diagonal (i.e., front-to-side or rear-to-side) beams to communicate with vehicles in other neighboring lanes, as shown in Figure 1b, rather than just side beams to reduce the collision risk between lanes. These diagonal transmissions are required while changing lanes in the convoy model or while communicating with other convoy vehicles.

### 3.1. GiV2V Communication Antenna

**Figure 2:**
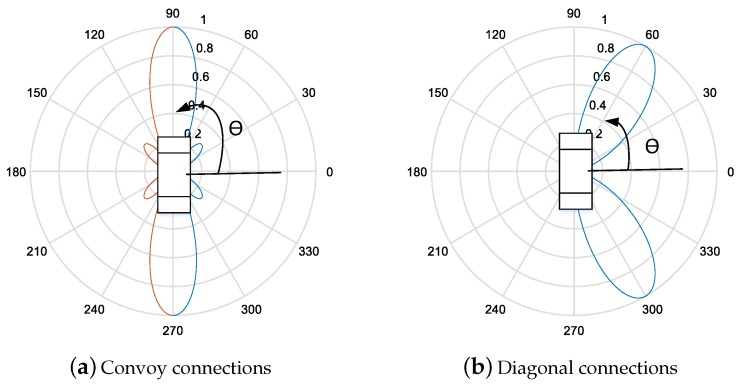
Beam direction and gain of linear array antenna (ULA).

For the beam alignment in the GiV2V network, the IEEE 802.11ad antenna module is required to support two types of beam patterns, as shown in Figure 1. Thus, we adopted a commercial IEEE 802.11ad product supporting beam patterns in our testbed, as illustrated in Figure 2. The directional antenna allows radiation intensity in a designated direction (θ,ϕ) with width of $\theta_{w}$ at a given transmission power in contrast to the omnidirectional antenna that emits the power in isotropic mode; θ and ϕ are the angles in z-axis [0, π) and xy-axis domains [0, 2π), respectively. Therefore, the directional antenna is useful for mmWave communications that suffer from high attenuation along the path. Furthermore, unlicensed bands at 60 GHz for WiGig allow low transmission power, only 10 dBm to avoid reciprocal interference between devices; thus, the antenna gain from the beam forming is important to increase the radio range.

The directional antenna gain is

$$g\left(\theta ,\phi \right)=\eta \frac{u(\phi ,\theta )}{u_{0}},$$

where η is the antenna efficiency with loss (0<η≤1) and $u_{0}$ is the average power density transmitted in all directions.

If we assume that the omnidirectional transmission power is nearly constant, then $u_{0}=1/4\pi P_{t}$ and the directivity $D=4\pi u\left(\theta ,\phi \right)/P_{t}$ if the loss η is negligible.

(1) $$g\left(\phi ,\theta \right)=\frac{\left|u(\phi ,\theta )\right|}{1/4\pi \int_{0}^{2\pi }\int_{0}^{\pi }\left|u\left(\phi ,\theta \right)\right|sin\left(\theta \right)d\theta d\phi }.$$

The beamforming gain g(θ,ϕ) of the a uniform linear array antenna (ULA) or uniform circular array antenna (UCA) is derived from Equation (1). The ULA antenna gain for beam directions is shown in Figure 2. The beam-width is the narrowest in the orthogonal direction (i.e., $\pm 90^{\circ }$) of bore sight and widest at $\pm 0^{\circ }$ when the antenna array is aligned at zero. Conversely, the UCA antenna can consistently form the same beam width at each sector.

The beam patterns shown in Figure 2 can be dynamically applied to the GiV2V topologies illustrated in Figure 1. For the convoy model of vehicles, the beam pattern of Figure 2a is appropriate while that of Figure 2b is suitable for the diagonal topology. Typically, the IEEE 802.11ad module explores all the possible sectors for beam directions provided by the RF front-end and antenna arrays. This type of beam sweeping procedure follows the IEEE 802.11ad standard. Furthermore, the vehicles can utilize the vehicular topology information to decide a beam direction. Assuming that all vehicles exchange hello messages (e.g., cooperative awareness message (CAM)) with neighbor nodes and recognize their location by GPS information, vehicles can choose a beam direction without sweeping or by at least reducing the number of sweeping sectors.

### 3.2. GiV2V Communication Radio Range

In this section, we introduce a well-known pathloss model of IEEE 802.11ad to compare the theoretical outcomes with the measurement results. Based on this model, we calculated the theoretical coverage value with the given system parameters of a commercial IEEE 802.11ad module (Section 4.1) and then compared it with measurement results (Section 5). Maltsev et al. [54] presented an indoor pathloss model of 60 GHz WLAN (i.e., WiGig) for the LOS environment based on the measurement study, as presented below.

(2) $$L_{d}\left(dB\right)=A+20log_{10}\left(f_{MHz}\right)+10\alpha log_{10}\left(d\right),$$

where the *A* is 32.5 dB and without a shadow factor. *d* is the distance between the transmitter and receiver (*km*) and α is a pathloss exponent of LOS (e.g., 2).

In outdoor GiV2V communication, additional attenuation from water vapor ($L_{vap}$), oxygen ($L_{O_{2}}$), and rain ($L_{R}$) are considered [8]. Those atmospheric parameters (dB/km) for further loss are assumed as a constant for the relatively short communication period in this study.

$$L_{a}\left(dB\right)=d\left(L_{vap}+L_{O_{2}}+L_{R}\right).$$

Accordingly, the total pathloss can be $PL\left(d\right)=L_{d}+L_{a}$. For simplicity, we assumed that the pathloss, $L_{a}$, owing to the atmospheric condition was static during the short communication period. Therefore, the pathloss was only determined by the distance, *d*, at a given operational frequency (e.g., 60 GHz).

In the LOS environment, the radio range can be determined by the following outage probability with the sensitivity level, *T*, of the target modulation coding scheme (MCS).

(3) $$P\left(P_{r}\geq T\right)=P\left(PL\left(d\right)\leq P_{t}+G_{R}+G_{T}-T-IL\right),$$

where IL is the implementation loss, such as that from cables. The maximum values of antenna gain of the transmitter $G_{T}$ and receiver $G_{R}$ are assumed to be the same when both have beam directions toward each other and use same number of antenna array.

The maximum GiV2V coverage, *d*, can be derived as $PL^{-1}\left(P_{t}+G_{R}+G_{T}-T-C\right)$. Therefore, the reachable probability between vehicles can be defined as P(D≤d) with a random variable, *D*, indicating the distance between a transmitter and receiver. In the above equation, the effective range is decided from only the antenna gain of the transmitter and receiver (i.e., beam forming factor) while the other component is the constant loss, *C*. In this study, we assumed no transmitter power control was achieved between vehicles. Using Equation (3), the maximum range, *d*, can be expressed as follows:

(4) $$d={\left(\frac{P_{t}G_{R}G_{T}}{TC}\right)}^{1/\alpha }.$$

## 4. Experimental Configuration

### 4.1. IEEE 802.11ad

Previously, the mmWave technologies were studied and standardized for WLANs, which are primarily used for home appliances and hand-held devices at indoor environments. IEEE 802.15.3 task group 3c (TG3c) [55] and IEEE 802.11ad [56] standards specify physical and MAC layer protocols at 60 GHz unlicensed bands. The IEEE 802.11ad defines operations between an access point (AP) and a mobile stations (STA) using a two-phase beam training (i.e., association beamforming training (A-BFT), beam refinement protocol (BRP)), in which both detect a transmitting or receiving sector approximately by sweeping all directions during the beacon header interval (BHI); subsequently beam refinement is performed by BRP during the service period (SP) of data transmission interval (DTI). Additionally, IEEE 802.11ad supports a relay mode for link blockage between the AP and the STA. During the SPs that are set by the AP for searching a relay STA, a source and destination STA exchange the BRP packets with the neighboring candidate relay STAs nearby. Subsequently, the source STA requests measurement reports from several possible relays with good channel quality (i.e., high signal to noise ratio (SNR)), which include the link state information to both the source and destination STAs. Then, the source STA finally selects the best relay that has the highest SNR in both links.

For the GiV2V testbed used in this study, we used off-the-shelf modules of IEEE 802.11ad [56]. The IEEE 802.11ad standard supports mmWave communications at 60 GHz unlicensed bands and was developed for a considerable time after the completion of the standard. The IEEE 802.11ad standard defines the specification of a physical and MAC protocol for an Access Point (AP) and a directional multi-gigabit (DMG) mobile station (STA). In addition, the relay DMG STA (RDS) operation is specified for the NLOS environment. IEEE 802.11ad has six channels with 2.1 GHz bandwidth each from 58.32 to 69.12 GHz spectrum, which allow different modulations with single or multiple carriers; the single carrier can achieve data rate from 385 Mbps to 4.62 Gbps and multiple carriers (such as orthogonal frequency-division multiplexing(OFDM)) can achieve data rate from 693 Mbps to 6.75 Gbps according to the MCSs.

**Figure 3:**
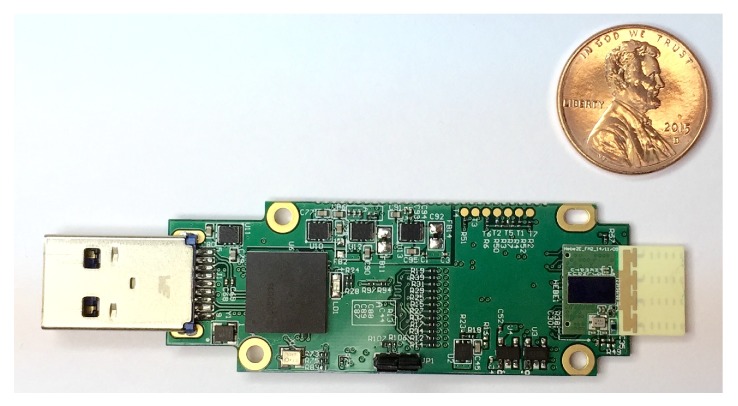
Tensorcom 802.11ad module for evaluation kit. [57].

In this study, we utilized the commercial products developed by Tensorcom [57] shown in Figure 3, which achieves the physical/MAC protocol stack of IEEE 802.11ad and are being used for communications between smart phones, televisions, laptops and their supplementary devices. The performance features of the Tensorcom 802.11ad module are noted listed in Table 2. With these parameters, expected theoretical radio range *d* can be calculated with a given pathloss exponent using Equation (4) from Section 3.2. The reachable distances according at the required threshold *T* = −68 dBm for MCS1 are presented in Table 3 with varying pathloss exponents.

### 4.2. On-Board Unit Installation

**Figure 4:**
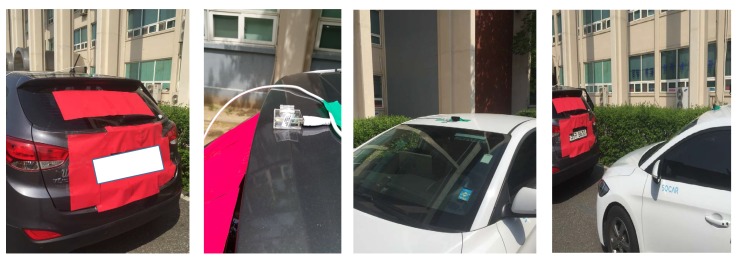
Testbed scenes.

We created on-board units (OBUs) for GiV2V communications using the Tensorcom IEEE 802.11ad modules and laptops (Intel Core i7 7700HQ, 4 GB memory, graphics processing unit) as host PCs. For a connection between the IEEE 802.11ad module and the host laptop, the Tensorcom 802.11ad module supports USB 3.0 interface; its physical data rate was 5 Gbps; however, the actual data rate measured on the packet level was less than 3 Gbps due to USB driver and Linux kernel overhead. The IEEE 802.11ad module processes physical and MAC protocol stacks for mmWave communications and the host PC deals with the upper layer protocols from an IP to identify the user datagram protocol (UDP)/transmission control protocol (TCP). The IEEE 802.11ad modules were installed on the vehicle roof and connected to Linux laptops via USB cables, as shown in Figure 4. In the host PC, an evaluation software was installed that generated UDP/IP packets to be sent to the IEEE 802.11ad module using the GNU USB library, and then the IEEE 802.11ad module encapsulated the MAC frames over those packets. Similarly, the 802.11ad module decapsulated the received MAC frames and forwarded the packets to the host. Additionally, the evaluation software calculated instantaneous throughput with the received packets and also allowed users to configure the beam directions, MCS levels and modes of intra-structure or ad hoc of the IEEE 802.11ad module. To collect information only available in the IEEE 802.11ad module such as SNR, receive signal strength (RSS), etc., control messages, such as SNR request and response, were defined to be exchanged between the IEEE 802.11ad module and host PC through the GNU USB library.

**Figure 5:**
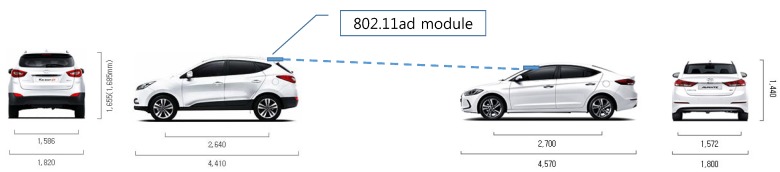
IEEE 802.11ad module installation on vehicles [58].

For our testbed, two vehicles were used to measure inter-vehicle connectivity of a mmWave link in the convoy model, as shown in Figure 1a. Thus, the front car had the WiGig module attached to the back end of the roof while the trailing car had another module attached to the front end of the roof, as shown in Figure 5. The front car (model: Tucsan ix) was a sport utility vehicle with a height 1.55 m and the trailing car (model: Elantra) was a sedan with a height 1.4 m, while their widths were similar. This installation nearly guaranteed LOS for the mmWave link while those two vehicles moved in the same lane.

### 4.3. Directional Antenna

In our experiment, the IEEE 802.11ad module was equipped with four end-fire antenna arrays (2 × 2 MIMO, two antenna arrays were used for reception and the other two antennas were used for transmission), which consisted of multiple short antenna arrays (an array is illustrated at the front-end, in the direction opposite to the USB host interface shown in Figure 3). In the end-fire antenna array, each antenna for the transmission or reception had different phases by 180 degree. There distance between the two antennas could be changed instead of the dynamic phase configuration performed in a phase array antenna. The Tensorcom 802.11ad module forms the beams toward the two different bore sights, as shown in Figure 2, for the transmission and reception using the two end-fire antenna arrays. With this, the module can sweep sectors to align the beam directions between the transmitter and receiver as described in the IEEE 802.11ad standard; accordingly, the module enables the configuration of beams dynamically according to vehicle positions on the roads.

### 4.4. Driving Test Environment

As the radio range is limited due to high pathloss of the mmWave channel, different driving environments on a campus and on city roads were considered to estimate the effect of vehicle speed and deceleration/acceleration.

**Figure 6:**
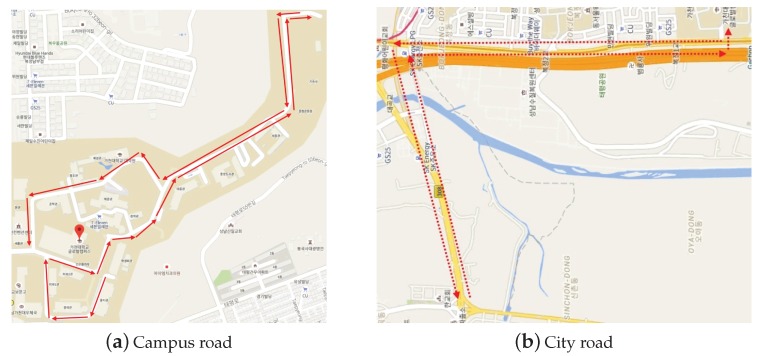
Campus and city routes for measurement.

Figure 6a shows the campus map of Gachon University, South Korea and the arrows in the map indicate the driving route for the measurement campaign, which was approximately 3 km. The measurement duration was approximately 400 s and the average speed was 27.7 km/h (campus speed limit is 30 km/h). A major part of the route consisted of sloping roads that could cause disconnection due to misaligned beam elevation. Further, the GiV2V connectivity was demonstrated on city roads, as shown in Figure 6b. The entire route was approximately 8 km and the speed limit was 80 km/h. There was no traffic during the measurement; however, 10 traffic signals for pedestrians and intersections existed along the route. For each stop, the acceleration and deceleration of the vehicles were repeated, which caused the vehicles to be apart by more than the reachable range specified by the 802.11ad mmWave link.

**Figure 7:**
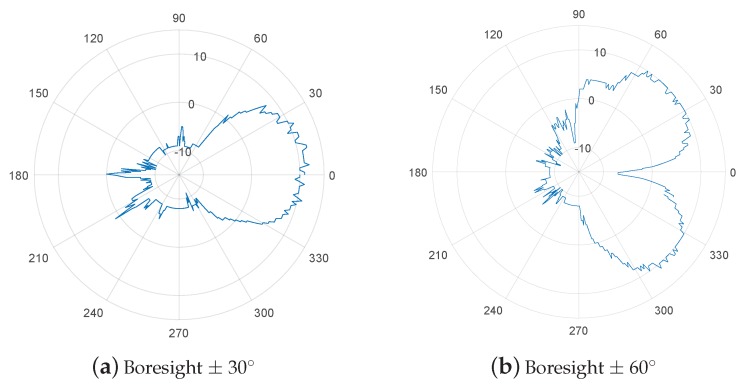
Measured beam patterns and SNR.

During the measurement time in both scenarios, two vehicles attempted to maintain a convoy model; however, the two vehicles were positioned in the diagonal direction, as shown in Figure 1, during a short period owing to lane change. Here, the beam form was selected between the two patterns shown in Figure 7, which follow the IEEE 802.11ad beam training procedure.

## 5. Experimental Result

### 5.1. Coverage and Beam Measurement

Prior to the driving experiment, we evaluated the performance of the IEEE 802.11ad module on static vehicles in terms of beam coverage and directions. For this measurement, the transmitter (i.e., vehicle) and receiver (i.e., laptop) were separated by 2 m, and continuous data traffic was transmitted from the transmitter to the receiver. The Tensorcom module supports only two sectors for beam forming, which covers ±30${}^{\circ }$ and 60${}^{\circ }$, respectively. We selected one of these sectors and transmitted dummy packets over the air. Then, we measured the received SNR in 360${}^{\circ }$ of directions around the transmitter.

Figure 7 shows the measured beam strength in SNR. The beam angle spreading over ±30${}^{\circ }$, as shown in Figure 7a, was appropriate for the convoy model of vehicles without interfering with other vehicles in adjacent lanes. Another beam sector covered ±60${}^{\circ }$, as shown in Figure 7b. The signal strength was the strongest at ±45${}^{\circ }$, where the two main lobes were created, while the SNR at approximately 0${}^{\circ }$ demonstrated a much lower value of −10 dB. This beam pattern was suitable for the diagonal connection model illustrated in Figure 1b. The SNR of the main and side lobes at the beam ±30${}^{\circ }$ case of the beam was approximately 12 dB and −10 dB, respectively. Similarly, the receiver SNRs of the main lobe and side lobe at ±60${}^{\circ }$ were approximately 11 and −10 dB, respectively. As the antenna directivity depends on the number of antennas in the array, the gain was comparable between the two beam patterns. The main lobe width was observed at nearly 60 degree in both patterns. The vehicles could switch between the two sectors according to change in the GiV2V topology.

**Observation** **1.** A pair of nodes using the IEEE 802.11ad standard with a directional antenna could achieve the required SNR only by the main-lobe and not by side-lobes.

**Figure 8:**
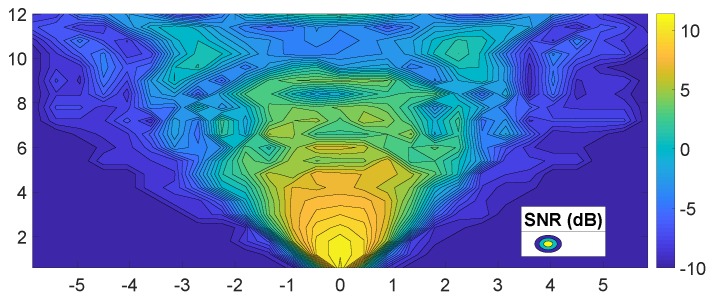
Measured SNR of beam angle ±30${}^{\circ }$.

We measured the received SNR of the boresight of ±30${}^{\circ }$ beam for varying distances between the transmitter and receiver. For distances within 5 m at the boresight, the SNR was more than 5 dB, while the SNR was approximately 0 dB at distances from 5 to 10 m, as illustrated in Figure 8. In areas farther than 10 m, the SNR varied with fading between −5 and −10 dB. Note that the SNR was less than −10 dB outside the beam angle.

**Observation** **2.** The gain difference between the main and side lobes was more than 15 dB in IEEE 802.11ad beam forming.

In addition, we measured the required SNR for each MCS level; we recorded the SNR values with which the 802.11ad module began to receive packets even with certain errors, while changing the distances between the transmitter and receiver nodes at each MCS level. According to Table 4, MCS 3 or 4 level was achievable at less than 5–6 m. After approximately 10 m, only MCS 0 or MCS1 was available. Table 5 lists the pathloss exponents calculated by the measured SNR values and approximate distances, as shown in Figure 8. The mean value of the pathloss exponents was 2.09, which was nearly same as a known pathloss exponent of the LOS. In addition, the measured SNR = −1 dB for MCS1 was available at approximately 10 m and its pathloss exponent was 2.1. Table 3 also indicates that the reachable distance could be approximately 10 m with the α = 2.1. Therefore, we can conclude that the theoretical calculation and measurement were almost identical, although there might be a little error in measurement.

**Observation** **3.** IEEE 802.11ad operating on 60 GHz demonstrates a pathloss exponent of approximately 2.1 at LOS.

**Figure 9:**
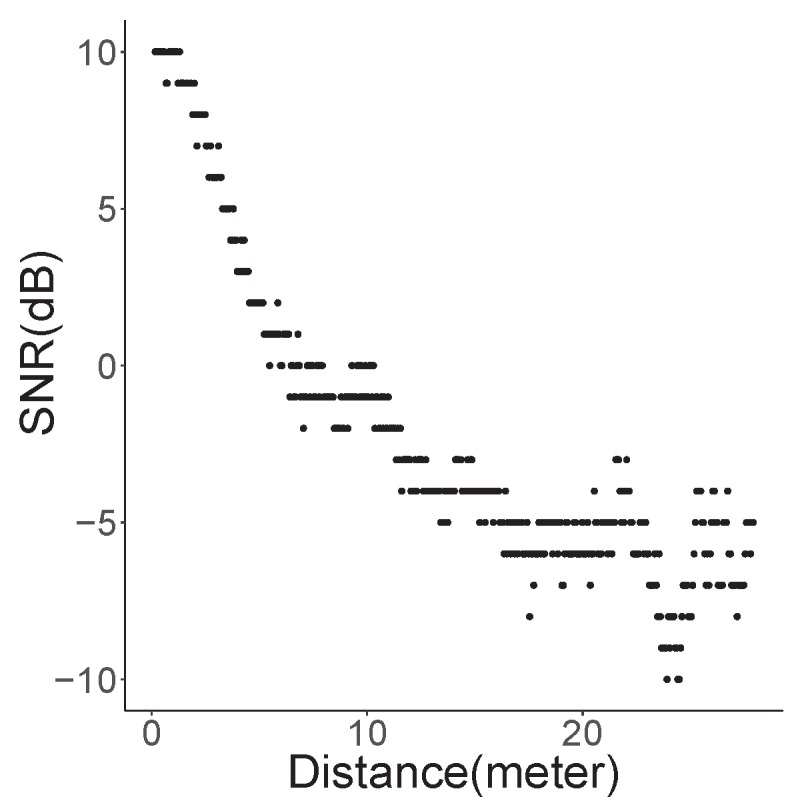
Received SNR of a slowly moving vehicle with beam angle ±30${}^{\circ }$.

Figure 9 shows the varying receive SNR values in the moving vehicle. In contrast to the previous measurement with fixed nodes, we obtained the received SNR samples from a slowly moving vehicle against a static transmitting vehicle. In this case, we observed that the SNR decreased exponentially corresponding to the distance; the SNR decreased by approximately 18 dB when the vehicle crossed the 20 m distance. By applying the pathloss equation (Equation (2)), $\alpha *10log(20/d_{0})=18$. Thus, the pathloss exponent was calculated as about 2.2 with the $d_{0}=3$, which is the distance between vehicles when the SNR is 10 dB. This measured exponent value was close to to the value of one of the LOS models described in [56].

**Observation** **4.** The receiver SNR fluctuates during the communication between moving vehicles.

### 5.2. Campus Experiment

**Figure 10:**
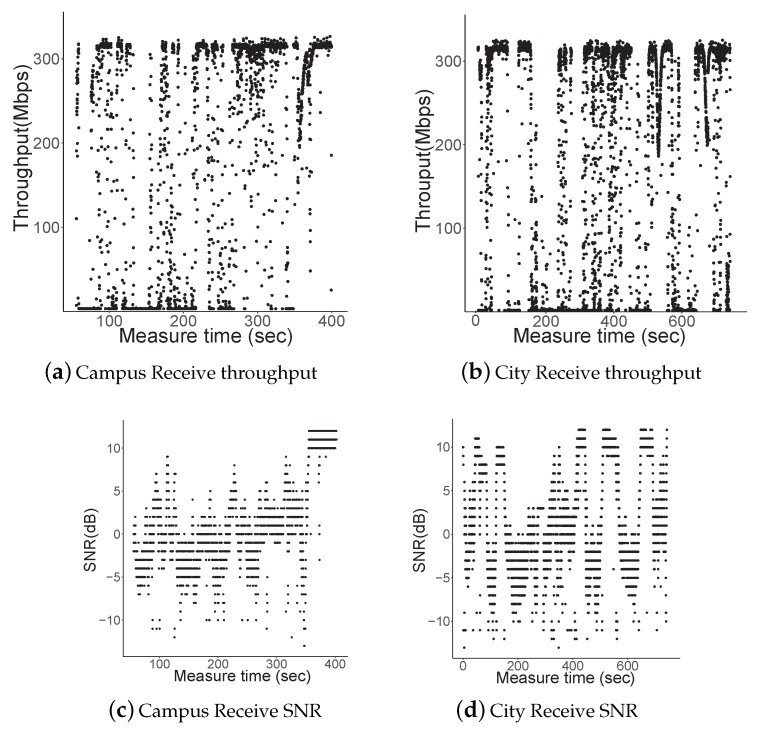
Campus and city road measurement results.

Figure 10 shows the measurement results while driving through the campus and on the city roads. The MCS level was configured statically as level 1 (maximum 385 Mbps), with which a receiver can receive approximately 2500 packets every 100 ms by MAC aggregation. Considering the vehicle length and inter-vehicle distance on the roads, we concluded that the higher MCS level of 3 or 4, which was achievable only when the distance between vehicles was within approximately 5 m, was not achievable considering the driving testbed. The throughput shown in Figure 10 was instantaneously derived in a link layer that included error detection and retransmission mechanism. A receiver calculated the throughput of correctly received packets every 100 ms.

In the measurement results of the drive through campus, which is illustrated in Figure 10a, the instantaneous throughput was widely scattered between 0 and 300 Mbps. Certain parts of measurement time demonstrated notable throughput. We observed that the throughput improved after 300 s while disconnections were observed clearly between 200 and 300 s. The corresponding SNR is plotted in Figure 10c. The average SNR increased marginally with time. Consequently, the required SNR for MCS 1 (i.e., measured value, −1 dB in Table 4) was satisfied within approximately 200–300 s.

To investigate the relation between inter-vehicle distance and received signal quality during the driving test, we attempted to estimate the inter-vehicle distance using a computer vision technique (i.e., detecting a vehicle object using a marker on the front vehicle, as shown in Figure 4, and estimating the distance from bird’s eye view) while measuring the signal quality of the received packets; however, it was difficult to couple a received signal sample from the IEEE 802.11ad module and calculated distance based on the dash-cam video because of the processing delay in the real-time video and the distance estimation error, although the disconnectivity pattern presented in the following sections demonstrated an approximate correlation with the estimated inter-vehicle distance. However, the inter-vehicle distance during driving could be inferred from the measurement results shown in Figure 8 and Figure 9.

**Figure 11:**
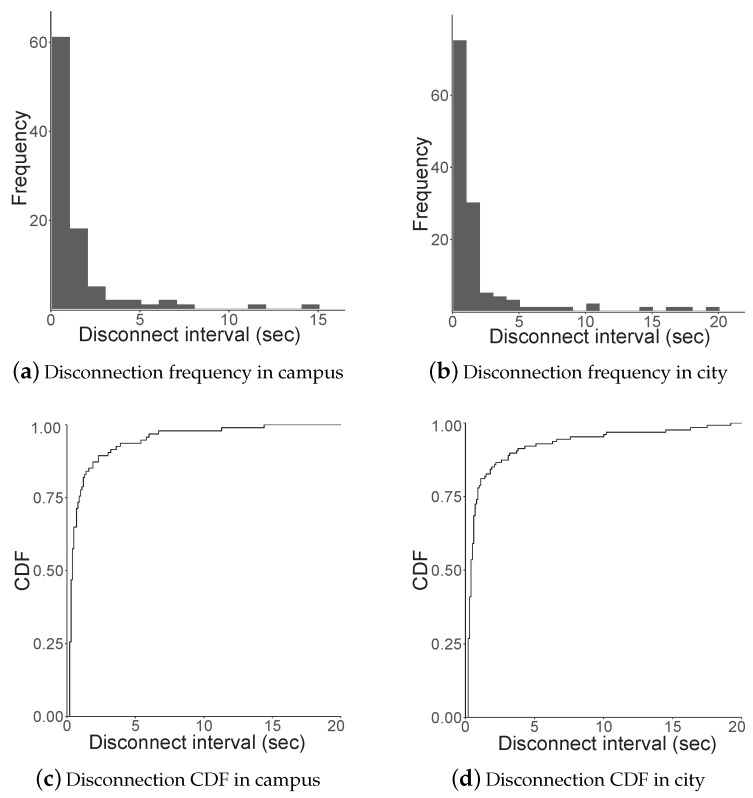
Campus and city road disconnectivity.

Figure 11 shows the disconnection interval and its probability. A disconnection interval is a period when a receiver cannot receive packets, i.e., the interval of samples with non-zero values. Our sampling interval could impose an error deviation of up to 100 ms. Most of the intervals existed for less than 2–3 s, as shown in Figure 11a. The cumulative distribution function (CDF) of the interval in Figure 11c shows that almost 90% of disconnection periods were less than 5 s.

**Observation** **5.** The SNR and connectivity in IEEE 802.11ad communications of slow-moving vehicles were more stable when compared to the fast-moving vehicles.

### 5.3. City Experiment

To investigate the GiV2V connectivity during high mobility, we performed experiments on normal city roads using the same configuration that was applied in the campus case. Figure 10b,d shows the results of the throughput and SNR while driving through the city, respectively. Here, we observed that disconnectivity occurred during the entire period of measurement because of mobility, while the connectivity in the campus measurement achieved partially stable periods. Owing to the 80 km/h speed limit and the deceleration/acceleration of vehicles on the city roads, the inter-vehicle distance varied more when compared to the campus experiment. The corresponding SNR plot in Figure 10d shows that the average SNR fluctuated more aggressively than the campus results. The campus SNR results in Figure 10c show that the SNR was stable because the vehicle convoy consistently maintained a low vehicle speed. However, samples with peak SNR (approximately 10 dB) were demonstrated more in the city measurement compared to the campus measurement because the vehicles on the campus almost did not stop during the test (approximately 400 s in Figure 10c; vehicles only stopped to end the test), while vehicles stopped at several intersections on the city road. Therefore, the measurement time with the peak SNR shown in Figure 10d indicated that the test vehicles stoppred at the intersection.

Further, as shown in Figure 11, the disconnectivity while traveling through the city roads was higher than that in campus owing to the variation in inter-vehicle distance. For instance, the longest disconnection period was approximately 20 s when compared to the 15 s in the campus case because the fast moving vehicles could not move closer once they were sufficiently separated to cause a loss of connection until the vehicle in front had slowed down to stop for traffic signals. Therefore, 90% of disconnection intervals was less than 10 s in Figure 11d, which was double the value of the campus result.

**Observation** **6.** Ten percent of disconnection period was more than 10 s in IEEE 802.11ad based V2V communications on city roads.

## 6. Discussion

**Figure 12:**
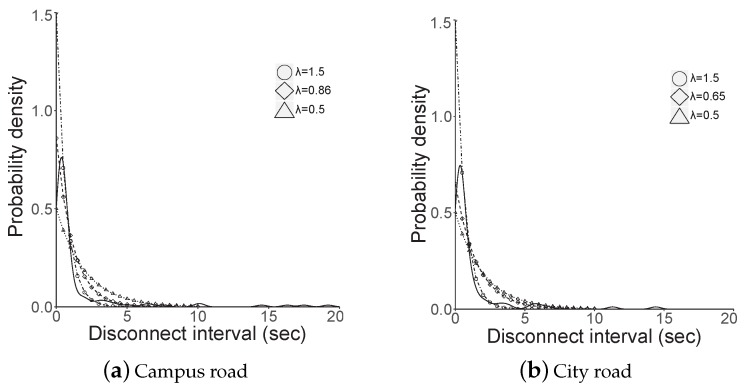
Probability density of disconnection.

According to the measurement results, the GiV2V connectivity differs from the driving environment and the degree of mobility affects the disconnection duration. Wireless communication parameters, pathloss and slow/fast fading of the GiV2V can be affected typically by varying vehicle speed in the different driving environment, but other parameters except the pathloss can be negligible considering the short radio range of the WiGig. However, the probabilities of disconnectivity in two different driving environments are compared, as shown in Figure 12. This figure illustrates the probability density function (PDF) of the disconnection interval in the campus and city measurements. As the probability of disconnectivity followed an exponential distribution, we inputed the measured values to the exponential distribution using the least-square error (LSE) minimization. The λ of the exponential distribution (i.e., 1/μ) was 0.65 and 0.86 for the campus and city roads, respectively; the expected intervals (μ) for the campus and city were 1.16 and 1.54 s, respectively. Although certain disconnectivity intervals were exceptionally longer in the city case, we can still argue that the average disconnection was comparable, at 1.1 and 1.5 s for campus and city measurements, respectively.

**Figure 13:**
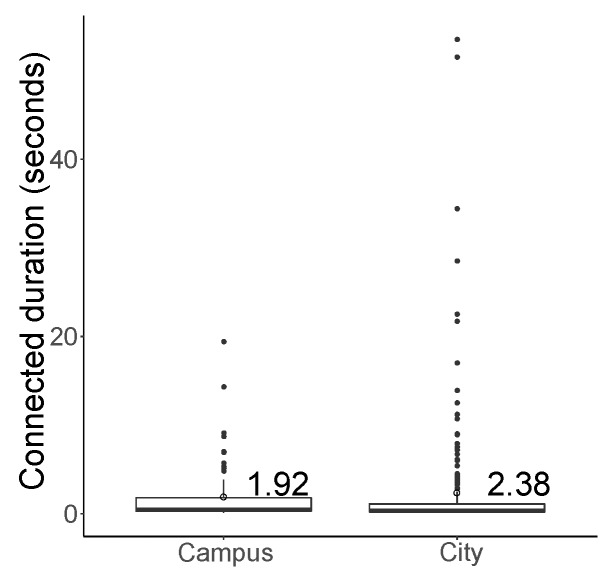
Average connection time while travelling through the campus and city.

Based on the results on connectivity duration shown in Figure 13, the maximum duration of maintaining a connection was 19.4 s in the campus test, while it was 53.5 s in the city scenario because vehicles stayed close at intersections for a long time owing to the traffic signal. Thus, the arithmetic mean of the connectivity time was higher in the city case (2.38 s) than the campus case (1.45 s). However, the value of geometric mean of the campus and city experiments were 0.7 and 0.6 s, respectively, and the median value of each was 0.5 and 0.4 s, respectively; the average connectivity duration in the campus scenario was higher than the city case, except during several long connectivity durations.

**Observation** **7.** The IEEE 802.11ad-based V2V links provided short connection of 1–2 s irrespective of the vehicle speed.

We list the summary of the experimental results of V2V communication using IEEE 802.11ad in Table 6. Based on the results, we concluded that the short radio range of IEEE 802.11ad caused too frequent disconnections even at slow vehicle speed. Moreover, the connectivity occurred differently corresponding to the driving environment; in campus, the vehicles demonstrated intermittent connections continuously, while vehicles demonstrated long connections only at intersection. However, the durations of the connections on the two roads were comparable and they allowed vehicles to deliver more data to each other. For instance, a vehicle could transmit approximately 300 MB during the short average connection time, 2 s.

However, those intermittent connections require more efficient and intelligent transport layer protocols and buffer management for retransmission and packet reordering. The conventional TCP congestion and flow control can suffer from retransmission of lost packets and connection management. Thus, light-weight protocols such as UDP with control signaling are probably appropriate and redundancy by fountain or network coding is advantageous for a reliable transmission.

Compared to our convoy experiments on the same lane, the disconnectivity interval becomes longer and the vehicle could not receive data continuously from the same transmitter if the vehicle moved individually without platooning. In this scenario, delay tolerant communication and data duplication on vehicular caches are necessary, where a receiver vehicle losing a transmitter due to long disconnection can request the data from a neighboring vehicle having duplicated data. As a future work, we will investigate distributing large data in a vehicular cloud using the GiV2V mmWave links and vehicular storages in the delay tolerant networking.

## Figures and Tables

**Table 1:** Survey on mmWave-based V2X communications.

References	Contents of the Studies on mmWave V2X Communications
[12,13,14,15,31,32,33]	Vehicular channel models with link blockage, scattering, shadowing and multipath fading are established. Furthermore, reflection and diffraction of mmWave at realistic road surfaces and geometries are explored for the model.
[34,35,36,53]	A stochastic model for V2X communications with blockage probability from RSU and throughput is presented.
[44,45,46]	Blockage detection and relay/multi-hop routing in V2X communications to avoid obstacles or extend network coverage is demonstrated.
[37,38,39,40,41,42,43]	Location or situation-based channel estimation, beam direction steering and training are achieved when prior channel information or past measurement is given for each location or situation. Machine learning techniques can be applied.
[18,19]	A mmWave link is configured using Long-Range Radar (LRR) mounted on the road infrastructures and on the vehicles for V2I and V2V communications.
[16,17]	The mmWave link configuration is assisted by motion and posture information of vehicles estimated from vehicular sensors. DSRC beacons carry the sensor information periodically.
[8,47]	Effect of inter-beam interference in V2V networks is analyzed and beam alignment and multi-channel assignment are considered.
[20,21,22]	Distributed beam alignment and width decision are achieved by channel and queue state information. V2V association and scheduling problem are solved in a decentralized manner.
[48,49]	Multi-connectivity with use of microwave frequencies (e.g., DSRC, LTE) is considered to increase the robustness connectivity (e.g., handover or relay) and reduce the beam tracking overhead.
[10,11,30]	Testbeds for mmWave V2I or V2V communications and experiment results are introduced.
[52]	The IEEE 802.11ad standard (WiGig) is investigated for mmWave V2X communications using simulation.
[50]	Security techniques of physical layer in mmWave and MIMO systems are proposed.

**Table 2:** Tensorcom 802.11ad module features.

EIRP	12.5 dBm
IL	7.5 dB
G	7.5 dBi
MIMO	2 × 2
BW	1.76 GHz
MCS	0–7

**Table 3:** Reachable distances according to pathloss exponents at MCS1.

Pathloss exponent (α)	2.0	2.1	2.2	2.3	2.4	2.5	2.6	2.7	2.8	2.9
Distance (m)	10.01	9.96	8.11	7.41	6.81	6.31	5.88	5.50	5.18	4.89

**Table 4:** Measured SNR against MCS level.

MCS	0	1	2	3	4	5	6	7
SNR (dB)	−10	−1	2.5	4.5	6	7.5	8.5	9.5

**Table 5:** Pathloss exponent calculation based on SNR measurements.

SNR (dB)	−1	2.5	4.5	6	7.5	8.5	9.5
Distance (m)	10	8	6	5	4	3.5	2.5
Pathloss exponent (α)	2.1	2.07	2.09	2.0	2.08	2.1	2.2

**Table 6:** Experimental results on driving test.

	Campus	City
Mean throughput (Mbps)	177	194
Mean disconnection (s)	1.16	1.54
Mean connection (s)	1.45	2.38
